# Using Body Composition Groups to Identify Children and Adolescents at Risk of Dyslipidemia

**DOI:** 10.3390/children8111047

**Published:** 2021-11-13

**Authors:** Alina Ofenheimer, Robab Breyer-Kohansal, Sylvia Hartl, Otto C. Burghuber, Florian Krach, Frits M. E. Franssen, Emiel F. M. Wouters, Marie-Kathrin Breyer

**Affiliations:** 1 Ludwig Boltzmann Institute for Lung Health, 1140 Vienna, Austria; robab.breyer-kohansal@leadstudy.at (R.B.-K.); sylvia.hartl@leadstudy.at (S.H.); otto.burghuber@leadstudy.at (O.C.B.); emiel.wouters@leadstudy.at (E.F.M.W.); marie-kathrin.breyer@leadstudy.at (M.-K.B.); 2 NUTRIM, Maastricht University Medical Center, 6200 MD Maastricht, The Netherlands; fritsfranssen@ciro-horn.nl; 3 Department of Respiratory and Critical Care Medicine, Clinic Penzing, 1140 Vienna, Austria; 4 Medical School, Sigmund Freud University, 1020 Vienna, Austria; 5 Department of Mathematics, ETH Zürich, 8092 Zurich, Switzerland; florian.krach@math.ethz.ch; 6 Department of Research and Education, CIRO, 6085 NM Horn, The Netherlands

**Keywords:** dyslipidemia, cardiovascular risk, physical activity, body composition, muscle mass, body compartments

## Abstract

The impact of body composition on the early origin of chronic diseases is an increasingly appreciated phenomenon. Little is known about the characteristics of children with varying body composition. The aim of this study was to investigate serum lipid profiles and other characteristics in relation to body composition. The data of 1394 participants (aged 6 to <18 years) of the observational general population-based Austrian LEAD Study have been analyzed. Body composition groups were defined by appendicular lean mass (ALMI) and fat mass (FMI) indices assessed by DXA. Serum lipid profiles (triglycerides, LDL-c, HDL-c) and other characteristics (e.g., prematurity, smoke exposure, physical activity, nutrition) were investigated in these body composition groups. Different body composition groups, which are not distinguishable by BMI, exist. Children with high ALMI and high FMI showed higher triglycerides and LDL-c, but lower HDL-c levels. In contrast, levels did not differ between those with high FMI but low (or normal) ALMI, and other body composition groups. BMI should be interpreted cautiously, and body composition should be measured by more precise techniques. In particular, children and adolescents with high FMI who have concomitantly high ALMI should be followed closely in future studies to investigate whether they are at increased risk of cardiovascular problems.

## 1. Introduction

The increasing prevalence of increased weight and obesity in children and adults worldwide imposes a tremendous burden on global health and economies [1]. The large increase in their prevalence in children is of great concern, since individuals being overweight or obese at a young age carry a higher risk for the early development of chronic diseases, including cardiovascular, metabolic, and respiratory diseases [1,2,3,4]. Commonly, body mass index (BMI, kg/m^2^) is used to classify individuals into weight categories to detect these conditions of overweight and obesity. However, body composition can vary widely within a group of subjects sharing the same BMI [5]. In 1964, Forbes described two groups of obesity in childhood: one group with increased lean mass (LM) additionally to high fat mass (FM); and a second group without an increase in lean mass [6]. Recent evidence suggests that the body composition group with high FM and low muscle mass is associated with greater health risks than either compartment alone [7,8]. Therefore, the amount of appendicular lean mass (ALM), a marker for skeletal muscle mass, might be important to consider when screening for early diseases. 

The lack of differentiation between various components of body weight might be the reason for the conflicting results of studies investigating the impact of BMI alone on health conditions [9]. There is increasing awareness that prenatal and early-life represent important periods for development, and several traits contributing to the development of body composition have been proposed [10,11,12]. The origins of atherosclerosis, diabetes mellitus type 2, and chronic respiratory diseases are also believed to be partly situated in utero or early infancy [13,14,15,16]. It has been shown that incidence of dyslipidemia at young age, especially in overweight and obese adolescents, is associated with increased carotid intima–media thickness in adulthood, an early marker of atherosclerosis [17,18]. Despite the increasing awareness of its importance, little is known about the various body constitutions in relation to risk profiles in children and adolescents. 

The aim of this study was to define body composition groups by appendicular lean mass and fat mass amounts assessed by dual-energy X-ray absorptiometry (DXA) in different BMI categories and to investigate their serum lipid profiles and early life risk factors (preterm birth, no breastfeeding), smoke exposure (second-hand, maternal), and lifestyle characteristics (physical activity, nutrition, socio-economic status).

## 2. Materials and Methods

### 2.1. Study Design and Population

The data were sampled by the Austrian LEAD (Lung, hEart, sociAl, boDy) Study (ClinicalTrials.gov; NCT01727518, https://clinicaltrials.gov/, accessed on 12 November 2021), a longitudinal, observational, population-based cohort study, representative of the general Austrian population. Inhabitants were randomly recruited based on the inhabitants’ register. More details about the study design, methodology, and the representativeness of the LEAD study cohort can be found elsewhere [19]. For the present analysis, cross-sectional data of participants aged 6 to <18 years, examined between 2011 and 2019, were included. Further inclusion criteria were valid DXA scans (verified by a trained study nurse) and availability of blood samples. The LEAD study was approved by the local ethics committee in Vienna (protocol number: EK-11-117-0711), and the study participants signed for their informed consent. For all minors, it was signed by their parents or legal representatives. 

### 2.2. Measurements

All study participants were examined in the study center after a fasting period of 8 h. A team of trained study nurses ensured the quality of data assessment. Body height was measured by a stadiometer and body weight by a high precision scale (exacta CLASSIC by SOEHNLE^TM^). BMI was calculated as weight (kg) divided by height^2^ (m^2^). Waist circumference was measured in the standardized fashion. Handgrip strength was measured on the dominant hand with a hand dynamometer (Trailite, TL-LSC100^TM^, LiteXpress GmbH, Ahaus, Deutschland); three successive measurements were performed and the highest value was kept. Whole-body scans were obtained by a Lunar Prodigy^TM^ (GE Lunar Corp.; Madison, WI, USA) DXA and analyzed with enCORE^TM^ (version 17, 2016). The coefficient of variation has been reported previously and found to be similar to other studies [20]. Body compartments should be normalized for total body height, hence it was essential to use the right index considering that height squared may not work best in children [21,22,23]. We recently confirmed that the exponents 2.5 and 3.5 account best for body height in FM and ALM, respectively [24]. Therefore, ALMI was calculated as the sum of the lean mass of all four limbs (kg) divided by height^3.5^ (m^3.5^), and FMI as fat mass (kg) divided by height^2.5^ (m^2.5^). 

Venous blood samples were collected, and total cholesterol, high-density lipoprotein cholesterol (HDL-c), and triglycerides were measured by photometric enzymatic method (Siemens Dimension Vista 150^TM^, Diamond Diagnostics Inc, Holliston MA USA). Low-density lipoprotein cholesterol (LDL-c) was calculated with the Friedewald formula. 

### 2.3. Definition of Variables

*BMI categories.* Reference values of the WHO were applied to calculate BMI z-scores. Study participants were classified into the following BMI categories according to their BMI z-scores: extreme thinness was defined as z-scores <−2, thinness as z-scores ≥−2 but <−1, overweight as z-scores >1 but ≤2, obesity as z-scores >2 and normal as z-scores ≥−1 but ≤1 [25].

### 2.4. Body Composition Groups

The cut-offs for the FMI and ALMI group categories low, normal, and high were set to the 25th and 75th age- and sex-specific percentiles. Low FMI and ALMI were defined as ≤25th percentile, normal as >25th but <75th percentile, and high as ≥75th percentile. The following combined body composition groups were assigned: high ALMI–FMI, low ALMI–FMI, low ALMI–high FMI, and high ALMI–low FMI. The normal FMI–ALMI group was defined as FMI >25th but <75th percentile and/or ALMI >25th but <75th percentile.

### 2.5. Blood Samples

Altered levels of total values were defined by using the cut-offs recommended by the American Academy of Pediatrics (AAP) guidelines: ≥100 mg/dl for triglycerides in children aged 6 to ≤9 years; ≥130 mg/dl for triglycerides in children >9 years; ≥130 mg/dl for LDL-c. A decreased HDL-c was defined as <40 mg/dl [26]. For each of the analyzed blood markers, an LMS model was further applied to construct smoothed percentiles accounting for age- and sex-specific differences. For analysis based on z-scores, values ≥95th percentile were defined as elevated, and for HDL-c values ≤5th percentile as decreased.

### 2.6. Questionnaires

All participants performed validated interviewer-based questionnaires, providing the following information. Socio-economic status (SES) was defined as a score based on education level, household income, and the occupational status of the parents or legal representatives. Three different categories of SES were defined: low as the 1st quintile (20th percentile); normal as quintiles 2 to 4; and high as the 5th quintile (80th percentile). Preterm birth was defined as birthweight <2500 g or gestational age <260 days. Second-hand smoking was defined by participants reporting to be or have been exposed to cigarette smoke during most days or nights. For maternal smoking, three categories were analyzed (ever smoking during, prior, or after pregnancy). Physical activity was calculated by the individuals’ reported number of minutes spent performing moderate- to high-intensity physical activity each day. Healthy nutrition was defined as drinking sugar-sweetened beverages less than daily and eating ≥2 portions of fruits and/or vegetables per day. 

### 2.7. Statistics

The sample was split into ALMI and FMI groups, and combined body composition groups, similar to the approach by Prado et al., were constructed [27]. The prevalence of each group was calculated for the total sample, as well as for males and females. The whole study cohort was divided into BMI categories, and the prevalence of the body composition groups was analyzed in each BMI category. Different characteristics were compared between all body composition groups. First, all parameters were evaluated if they corresponded to a normal distribution via the Kolmogorov–Smirnov test. For continuous, non-normally distributed variables, medians and quantiles 25 (q25) and 75 (q75) were calculated. For all continuous, normally distributed variables, means and standard deviations were calculated. To test for differences between the groups, Wilcoxon-rank sum test was performed. For all dichotomous variables, the prevalence was calculated, and group differences were tested by Fisher’s exact test. Multiple testing correction (Bonferroni–Holm) was applied to all *p*-values, and the significance level was set to 5%.

## 3. Results

Of the 1573 children and adolescents with valid DXA measurements, 1394 study participants had blood samples available. The sample size and distribution of ALMI and FMI groups are shown in Table 1.

**Figure 1:**
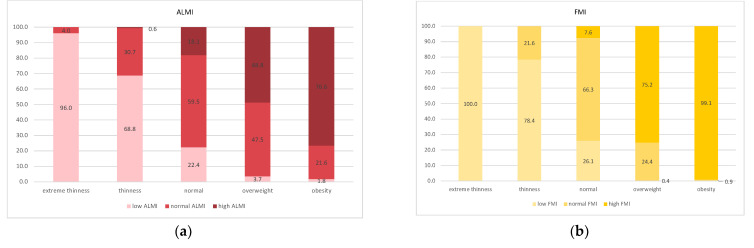
Prevalence of body compartments in BMI categories. Figure shows the prevalence of the different groups (low, normal, high) for FMI (**a**) and ALMI (**b**), respectively, in different BMI categories. BMI categories (extreme thinness, thinness, normal, overweight, obesity) were defined according to WHO. FMI and ALMI groups were defined by age- and sex-specific groups (low: ≤25th percentile, normal: >25th but <75th percentile, high: ≥75th percentile).

**Figure 2:**
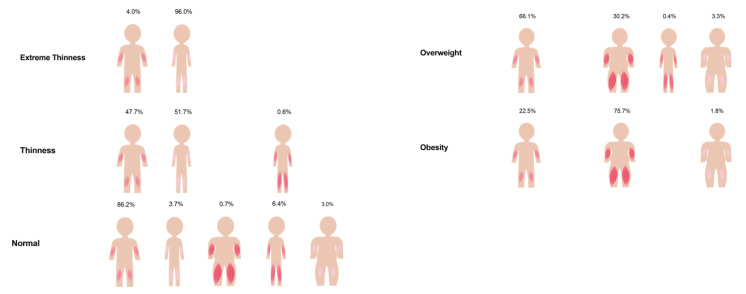
Prevalence of body composition groups in BMI categories. Figure shows the prevalence of the different body composition groups in BMI categories. The different body composition groups are represented by different figures. Varying amount of FMI are indicated by body shape (thin, middle, broad), ALMI amount by color shade (light, middle, dark) of displayed skeletal muscles mass in arms and legs region.

Among the body composition groups, the prevalence of the normal ALMI–FMI group was highest (71.3% overall), followed by the high ALMI–FMI (11.7% overall) and the low ALMI–FMI groups (10.5% overall). The low ALMI–high FMI group was prevalent in only 2.5%, and the high ALMI–low FMI group showed a prevalence of 4.0%. The prevalence was similar across all age groups (Table S1). Body composition data for the different BMI categories are illustrated in Figure 1. In the extreme thinness and thinness groups, the majority had low FMI and low ALMI values. Changes in the ALMI and FMI were masked in the normal BMI category. The prevalence of high ALMI and high FMI increased in the overweight and obese categories (Figure 1). In contrast to the sharp increase of high FMI from the normal to the overweight category (from 7.6% to 75.2%), the increase in ALMI was more gradual (from 18.2% to 48.8%). Figure 2 illustrates the prevalence of the body composition groups in the different BMI categories. Almost all extremely thin participants had low ALMI–FMI, while only half of the thinness group showed this body composition combination. In children and adolescents classified as normal according to their BMI, all different body composition groups were present. In participants classified as overweight, 30.2% had high ALMI–FMI values, which increased to 75.7% in the subjects with obesity. Isolated high FMI was present in a minority of these children and adolescents (3.3% and 1.8%, respectively).

### 3.1. Serum Lipid Profiles

**Figure 3:**
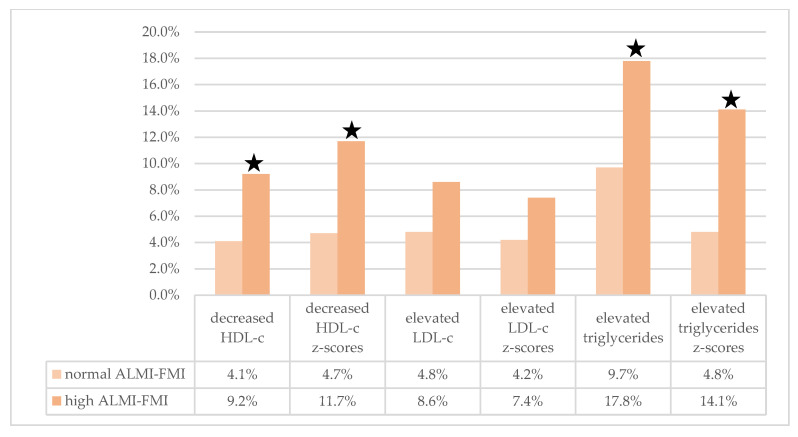
Prevalence of altered lipid profiles in the high ALMI–FMI and the normal ALMI–FMI groups. Figure shows the prevalence of elevated (for HDL-c decreased, respectively) serum lipid levels in the high. ALMI–FMI and the normal ALMI–FMI groups. Star indicates a *p*-value < 0.05 according to Fisher’s Exact test after applying Bonferroni–Holm multiple testing correction.

HDL-c was higher in the low ALMI and the low FMI groups, while levels were lower in the high FMI group (Table 2). In contrast, LDL-c and triglycerides were higher in children and adolescents with high FMI. The high ALMI–FMI body composition group showed marked differences in serum lipid levels. LDL-c and triglyceride levels were significantly higher in the high ALMI–FMI group. Contrary, HDL-c levels were significantly lower in this group. These differences persisted considering z-scores (which account for age and sex) of the serum lipids. Prevalence of decreased HDL-c and elevated triglycerides was higher in the high ALMI–FMI group compared to the normal ALMI–FMI group (Figure 3). Prevalence of altered serum lipids in all body composition groups are provided in the supplemental material (Table S2).

### 3.2. Body Composition Group Characteristics

While half of the children and adolescents with low FMI had a high SES, only 22.9% and 31.3% with high FMI had a high SES. Birthweight, preterm birth, and breast feeding did not differ significantly between body composition groups (Table 3). Only 8% of the high ALMI–FMI group were born preterm, while it was 15.1% in the low ALMI–FMI group. A quarter of those with high FMI reported second-hand smoking. This was found in both body composition groups with high FMI, independent from ALMI amount. The prevalence of children exposed to maternal smoking was also highest in the low ALMI–high FMI group, followed by the high ALMI–FMI group. Physical activity was higher in the high ALMI–low FMI group compared to the low ALMI–FMI, and the high ALMI–FMI group. Moreover, the prevalence of physical activity ≥60 min per day was significantly higher in the high ALMI–low FMI group compared to the low ALMI–FMI group (91.1% and 58.9%, respectively) and to the high ALMI–FMI group (91.1% and 54.0%, respectively). Between all other groups physical activity was similar. All other analyzed parameters did not show significant differences, and although hand grip strength was highest in the high ALMI–FMI group, it did not differ significantly.

## 4. Discussion

The present study found that BMI poorly reflects differences in body composition existing in childhood. It shows that low ALMI–FMI and high ALMI–FMI are the most frequent aberrant body composition groups. Early dyslipidemia was found in children and adolescents who have high FMI with simultaneously high ALMI values.

Those within the normal BMI category showed an especially huge variety of ALMI and FMI combinations. This has been previously shown in specific groups [28,29], but the present study extends this finding on a population-based level with data obtained by DXA. Even though BMI is widely used as a surrogate measure of fat mass mainly, we show that a close relationship exists also with ALMI, since the prevalence of high ALMI increased similarly with the BMI category, and was also highest in the obesity category (76.6%). Equally, FMI and ALMI both decreased by decreasing the BMI category. The present analysis shows that already at young age, different body composition groups exist within the general population, with similar distribution throughout childhood and adolescence. Longitudinal assessment in birth cohorts has shown that most children usually track along with stable BMI percentiles [30,31,32,33]. Our cross-sectional data suggest a comparable persistence for body composition patterns. 

Fetal and early postnatal programming is considered as an important factor for skeletal muscle development [34]. Aside from the normal ALMI–FMI group, the groups with high ALMI–FMI and with low ALMI–FMI showed the highest frequency. These results confirm that the body compartments LM and FM correlate positively with each other [20,35]. They further show that an increase in ALMI and FMI is dominant in overweight and obese children, and that an isolated increase in FMI only is rarely found.

Low muscle mass contributes to several adverse health outcomes, and, together with low muscle strength, is consistently associated with reduced bone parameters during growth, increasing the risk of osteoporosis at older age [8,36,37,38,39]. Sarcopenic obesity, which is the combination of high FM and low muscle mass, is associated with greater health risks [7,8]. In our cohort, this group with high FMI–low ALMI had a prevalence of 2.5% only. Our data stress the importance of body composition assessment in childhood and indicate that individual body constitution is probably determined early and often persists throughout life. Longitudinal data on body composition are needed to identify and specify body composition changes and to track low muscle mass.

Which traits determine body composition is not yet fully understood. In the present analysis, suggested early-life risk factors have been investigated, but no significant difference in any of these factors existed between the body composition groups analyzed. Besides genetics and early risk factors, nutrition and physical activity may also actively influence body composition [10,40]. While the prevalence of healthy nutrition was similar across all groups, reported physical activity differed. The high ALMI–low FMI group reported to spend more time doing physical activity. Considering that these parameters were assessed by questionnaire, they should be interpreted cautiously. Still, other studies, which measured physical activity objectively by accelerometer, support this result [40,41,42]. Future studies are needed to explore the role of protein intake in relation to the body composition groups analyzed. Indeed, higher protein intake is associated with higher fat-free mass (FFM), increased FM, and obesity risk [43,44,45]. Furthermore, it is not only the protein quantity but also the protein source which seems to influence muscle mass in childhood [43,46].

Intriguingly, the group of high ALMI–FMI showed marked differences in lipids compared to other groups. HDL-c levels were lower, while triglyceride and LDL-c levels were higher. A previous observational study in 660 adolescents (aged 16–17 years) reported that those with low muscle mass had significantly higher values of fasting triglycerides and atherogenic index, defined as the ratio triglycerides/HDL-c [47]. In contrast, study subjects with high ALMI of the present cohort values showed higher values of triglycerides, also after adjusting for age and sex. Another paper reported that in school children a greater hand grip strength was associated with healthier triglyceride and HDL-c concentrations, although these relationships were not independent of BMI [39]. In a cohort of 3320 participants (aged 5–18 years), body fat (assessed by skinfold thickness) ≥25% in males and ≥30% in females, was associated with cardiovascular risk factors [48]. Others confirmed that excess body fat (>37%) is associated with increased triglycerides, total cholesterol, LDL-c, and decreased HDL-c levels [49]. The prevalence of dyslipidemia was also reported to be significantly higher in U.S. youths with high adiposity. Prevalence varied from 12.4% (LDL-c) to 21.3% (triglycerides) in the high adiposity subgroup [50]. We found decreased HDL-c levels in the high FMI and the high ALMI–FMI groups. A negative association between HDL-c and ALMI has been previously reported [51,52]. Our study does not only support this finding, but confirms it by age- and sex-adjusted lipid values. Taking these factors into account is important, since FFM and muscle mass growth vary greatly by development stage [8]. Our data confirm that, total body weight and FMI amount in particular are associated with dyslipidemia in youth, but suggest that ALMI amount seems to additionally alter lipid levels to varying degrees. Hence, these findings stress the importance of early cardiovascular risk assessment, in particular for those with excess body fat. However, further longitudinal studies are required to demonstrate a link between this body composition group and cardiovascular risks as effects on pulse wave velocity. In that case, in-depth metabolic profiling is needed to explore possible underlying biological mechanisms.

A strength of the present study is that body compartments were assessed by DXA with very good reported accuracy and repeatability [53,54]. Additionally, FM and ALM were accurately normalized for body size; thus, biases related to body height were minimized as much as possible. Many studies did not consider that height squared often does not account for height correctly in children [21,22,23]. Another important strength is that our results were confirmed by age- and sex-adjusted lipid levels. To our knowledge, combined body composition groups have not been analyzed previously in such a large cohort of children and adolescents recruited from the general population. Our data underscore the need to use this approach in future studies and to consider the relationship between body compartments. Tracking these body composition groups longitudinally and investigating related traits could contribute fundamentally to understanding body composition development. However, some limitations have to be considered as well. First of all, cut-offs for ALMI and FMI groups were chosen arbitrarily due to the lack of validated cut-offs. Secondly, the reported data were collected in a single centre, recruiting Caucasian Middle-European youths. Thirdly, body composition data from birth to the age of 6 years were not available.

## 5. Conclusions

The results confirm that BMI does not reflect body constitution and we urge that the use of more precise assessment techniques should be emphasized for investigating body composition. In addition, children and adolescents with high ALMI–FMI showed an adverse cardiovascular risk profile. In the future, the prevalence of low muscle mass in these youths should be focused on. Prospective longitudinal data in the presented body composition groups are needed to define the impact of body composition on overall health across the lifecycle and to identify novel preventive strategies.

## Figures and Tables

**Table 1:** Sample size and prevalence of body composition groups and BMI categories.

	Males *n* (%)	Females *n* (%)	Overall *n* (%)
FMI (kg/m^2.5^)			
normal	348 (46.8%)	307 (54.1%)	655 (47.0%)
low	206 (27.7%)	177 (27.2%)	383 (27.5%)
high	190 (25.5%)	166 (25.5%)	356 (25.5%)
ALMI (kg/m^3.5^)			
normal	361 (48.5%)	333 (51.2%)	694 (49.8%)
low	185 (24.9%)	159 (24.5%)	344 (24.7%)
high	198 (26.6%)	158 (24.3%)	356 (25.5%)
ALMI-FMI groups			
normal ALMI-FMI	535 (71.9%)	459 (70.6%)	994 (71.3%)
low ALMI-FMI	72 (9.7%)	74 (11.4%)	146 (10.5%)
high ALMI-FMI	79 (10.6%)	84 (12.9%)	163 (11.7%)
low ALMI-high FMI	21 (2.8%)	14 (2.2%)	35 (2.5%)
high ALMI-low FMI	37 (5.0%)	19 (2.9%)	56 (4.0%)
BMI category			
extreme thinness	16 (2.2%)	9 (1.4%)	25 (1.8%)
thinness	83 (11.2%)	93 (14.3%)	176 (12.6%)
normal	443 (59.5%)	397 (61.1%)	840 (60.3%)
overweight	131 (17.6%)	111 (17.1%)	242 (17.4%)
obesity	71 (9.5%)	40 (6.2%)	111 (8.0%)
total	744	650	1394

Table shows sample size (*n*) and prevalence (%) of ALMI, FMI, combined body composition groups, and BMI categories. FMI = fat mass index, ALMI = appendicular lean mass index, BMI = Body mass index.

**Table 2:** Characteristics of serum lipid profiles in body composition groups.

	HDL-c [mg/dL]	HDL-c z-Scores	LDL-c [mg/dL]	LDL-c z-Scores	Triglycerides [mg/dL]	Triglycerides z-Scores
FMI [kg/m^2.5^]						
normal	61.0 (53.0, 70.0)	0.0 (−0.6, 0.6)	86.6 (70.4, 102.5)	0.0 (−0.7, 0.6)	63.0 (48.0, 86.0)	0.0 (−0.7, 0.6)
low	65.0 (54.0, 75.0) ^■^	0.3 (−0.3, 1.0) ^■^	82.4 (68.3, 97.3)	−0.2 (−0.8, 0.4)	57.0 (47.0, 77.0)	−0.3 (−0.8, 0.4)
high	54.5 (46.0, 64.0) ^■ †^	−0.4 (−1.2, 0.2) ^■ †^	92.8 (77.3, 111.3) ^■ †^	0.3 (−0.4, 0.9) ^■ †^	74.0 (56.0, 104.3) ^■ †^	0.3 (−0.3, 1.0) ^■ †^
ALMI [kg/m^3.5^]						
normal	59.0 (51.0, 70.0)	0.0 (−0.7, 0.6)	86.6 (71.9, 102.0)	0.0 (−0.6, 0.6)	62.0 (49.0, 86.0)	0.0 (−0.7, 0.6)
low	64.0 (54.8, 72.0) ^■^	0.2 (−0.4. 0.8) ^■^	84.2 (69.9, 102.9)	−0.1 (−0.8, 0.6)	62.0 (48.0, 86.3)	−0.1 (−0.7, 0.6)
high	58.0 (48.8, 69.0) ^†^	−0.1 (−0.9, 0.6) ^†^	90.2 (74.0, 108.4)	0.1 (−0.6, 0.8)	66.0 (51.0, 93.3)	0.1 (−0.6, 0.9)
ALMI-FMI groups						
normal ALMI- FMI	60.0 (52.0, 70.0)	0.0 (−0.6, 0.6)	86.7 (71.4, 102.0)	−0.0 (−0.6, 0.6)	62.0 (49.0, 87.8)	−0.0 (−0.7, 0.6)
low ALMI-FMI	66.0 (57.0, 75.8)*	0.4 (−0.3, 1.0)*	80.8 (66.9, 97.8)	−0.2 (−0.8, 0.5)	58.5 (47.0, 78.0)	−0.3 (−0.8, 0.4)
high ALMI-FMI	53.0 (45.0, 62.0) *^▲^●	−0.5 (−1.2, 0.0) *^▲^●	97.4 (77.6, 113.7) *^▲^	0.4 (−0.5, 1.0) *^▲^	76.0 (57.0, 106.0) *^▲^●	0.4 (−0.3, 1.1) *^▲^●
low ALMI-high FMI	60.0 (50.0, 64.5)	−0.2 (−0.8, 0.5)	92.0 (76.1, 112.1)	0.4 (−0.4, 1.0)	70.0 (54.0, 91.0)	0.3 (−0.4, 0.7)
high ALMI-low FMI	66.0 (53.8, 77.0)	0.4 (−0.4, 1.0)	84.3 (70.8, 96.2)	-0.1 (−0.7, 0.5)	57.5 (46.8, 69.2)	−0.4 (−0.7, 0.3)

Table shows medians and quartiles (q25, q75) for each continuous parameter not corresponding to a normal distribution (according to Kolmogorov–Smirnov test), and means ± standard deviation for normally distributed parameters (according to Kolmogorov–Smirnov test). *p*-values were calculated by Wilcoxon-rang sum test to test for differences between each group, and Bonferroni–Holm correction was applied. *Comparison between separate low, normal, high ALMI (FMI, respectively) groups*: † *p*-value < 0.05 vs. low ALMI (FMI, respectively), ■ *p*-value < 0.05 vs. normal ALMI (FMI, respectively). *Comparison between combined ALMI-FMI body composition groups*: * *p*-value < 0.05 vs. normal ALMI-FMI group. ▲ *p*-value < 0.05 vs. low ALMI-FMI group, ● *p*-value < 0.05 vs. high ALMI-low FMI group. HDL-c = high-density lipoprotein cholesterol, LDL-c = low-density lipoprotein cholesterol. FMI = fat mass index, ALMI = appendicular lean mass index.

**Table 3:** Characteristics of body composition groups.

	Normal ALMI FMI	Low ALMI FMI	High ALMI FMI	Low ALMI High FMI	High ALMI Low FMI
**Demographics**					
age [years]	10.8 (8.3, 14.6)	10.9 (8.7, 14.5)	10.8 (8.7, 14.5)	9.9 (8.5, 15.1)	10.4 (8.2, 16.1)
sex [%females]	46.2%	50.7%	51.5%	40.0%	33.9%
height [cm]	146.0 (132.0, 164.0)	151.5 (133.0,168.0)	148.0 (135.0,163.0)	146.0 (136.0,166.5)	140.5 (127.0,168.3)
weight [kg]	39.0 (28.0, 55.0)	33.5 (24.0, 46.8) *	54.0 (38.0, 71.0) *	43.0 (32.5, 60.0)	33.5 (25.8, 56.5)
waist circumference [cm]	65.5 (59.0, 74.5)	60.5 (55.6, 68.5) *	80.5 (70.8, 90.3) *	76.0 (66.3, 80.5) *	60.8 (56.0, 68.6)
hand grip strength [kg]	17.5 (12.9, 26.2)	18.2 (12.5, 26.5)	19.5 (14.2, 27.2)	13.2 (11.4, 21.9)	19.6 (13.9, 32.2)
Socio-economic status					
low	11.8%	8.2%	21.5%	8.6%	10.7%
normal	44.3%	39.7%	47.2%	68.6%	39.3%
high	43.9%	50.7%	31.3%	22.9%	50.0%
**Early life risk factors**					
birthweight [kg]	3.3 (2.8, 3.7)	3.2 (2.4, 3.6)	3.3 (2.7, 3.7)	3.3 (1.0, 3.9)	3.2 (2.3, 3.5)
preterm birth [%]	9.2%	15.1%	8.0%	11.4%	14.3%
low birthweight	20.7%	26.0%	22.1%	34.3%	28.6%
breast feeding ever	89.5%	91.1%	82.8%	82.9%	96.4%
**Smoke exposure**					
second-hand smoking	17.5%	15.1%	25.3%	25.7%	3.6%
maternal smoking					
prior pregnancy	38.2%	34.2%	44.2%	65.7%	26.8%
during pregnancy	9.3%	4.1%	14.7%	22.9%	3.6%
after pregnancy	11.9%	8.9%	16.6%	22.9%	5.4%
**Lifestyle factors**					
physical activity [minutes/day]	79.3 (49.8, 110.7)	70.7 (43.2, 103.6)	62.1 (41.4, 99.3)	70.7 (44.3, 98.3)	95.0 (77.9, 120.5)
physical activity ≥60 minutes/day	67.8%	58.9%	54.0% *	68.6%	91.1% *
healthy nutrition	26.9%	26.0%	28.2%	25.7%	26.8%

Table shows medians and quartiles (q25, q75) for continuous parameters, and prevalence (%) for dichotomous parameters for each body composition group. * *p*-value < 0.05 after Bonferroni–Holm correction compared to normal ALMI–FMI group.

## Data Availability

Data is contained within the article or supplementary material.

## Supplementary Materials

The following are available online at https://www.mdpi.com/article/10.3390/children8111047/s1, Table S1: Sample size and prevalence of body composition groups in different age groups. Table S2: Prevalence of elevated levels of serum lipids in body composition groups.
