# Supplementary material for: Using Body Composition Groups to Identify Children and Adolescents at Risk of Dyslipidemia
Source: Children (Basel). 2021 Nov 13;8(11):1047. doi: 10.3390/children8111047 (PMC8625256; doi:10.3390/children8111047)
Supplement: Supplementary file 1 [file children-08-01047-s001.zip › children-1424214-supplementary.pdf]

Supplemental Material

Table S1. Sample size and prevalence of body composition groups in different age groups

**both sexes**	**6-<10 years %**	**10-<14 years %**	**14-<18 years %**
normal ALMI-FMI	71.5%	72.9%	69.5%
low ALMI-FMI	9.4%	11.5%	11.1%
high ALMI-FMI	11.7%	12.0%	11.3%
low ALMI-high FMI	3.0%	1.3%	2.9%
high ALMI-low FMI	4.4%	2.3%	5.2%
**males**			
normal ALMI-FMI	70.1%	75.3%	71.0%
low ALMI-FMI	9.4%	10.2%	9.5%
high ALMI-FMI	11.0%	10.2%	10.4%
low ALMI-high FMI	3.6%	0.9%	3.6%
high ALMI-low FMI	5.8%	3.3%	5.4%
**females**			
normal ALMI-FMI	72.9%	69.9%	67.7%
low ALMI-FMI	9.4%	13.1%	12.9%
high ALMI-FMI	12.5%	14.2%	12.4%
low ALMI-high FMI	2.4%	1.7%	2.2%
high ALMI-low FMI	2.8%	1.1%	4.8%

Legend

Table shows sample size (n) in age groups of 4-years.

Table S2. Prevalence of altered levels of serum lipids in body composition groups

**parameter**	**normal ALMI FMI**	**low ALMI FMI**	**high ALMI FMI**	**low ALMI high FMI**	**high ALMI low FMI**
HDL-c <40mg/dl	4.1%	5.5%	9.2%	8.6%	3.6%
HDL-c z-scores ≤5th percentile	4.7%	4.1%	11.7%	8.6%	0.0%
LDL-c ≥130mg/dl	4.8%	6.2%	8.6%	5.7%	0.0%
LDL-c z-scores ≥95th percentile	4.2%	4.8%	7.4%	5.7%	1.8%
triglycerides ≥100/≥130mg/dl	9.7%	6.2%	17.8%	8.6%	7.1%
triglycerides z-scores ≥95th percentile	4.8%	4.8%	14.1%	2.9%	3.6%

Legend

Table shows the prevalence of elevated (for HDL-c decreased, respectively) serum lipid levels in different body composition groups.
